# Bochdalek hernia with intrathoracic spleen treated by robotic-assisted mesh repair utilizing indocyanine green contrast media intraoperatively. A case report

**DOI:** 10.1093/jscr/rjab352

**Published:** 2021-08-16

**Authors:** Alexandros Kozadinos, Dimosthenis Chrysikos, Spyridon Davakis, Ioannis Kozadinos, Panagiotis Farmakis, Georgios Georgiou, Theodore Troupis

**Affiliations:** Department of Surgical Anatomy, School of Medicine, National and Kapodistrian University of Athens, Athens, Greece; A’ Robotic and Minimal Invasive General Surgery Department of Metropolitan Hospital of Athens, Athens, Greece; Department of Surgical Anatomy, School of Medicine, National and Kapodistrian University of Athens, Athens, Greece; Department of Surgical Anatomy, School of Medicine, National and Kapodistrian University of Athens, Athens, Greece; A’ Robotic and Minimal Invasive General Surgery Department of Metropolitan Hospital of Athens, Athens, Greece; A’ Robotic and Minimal Invasive General Surgery Department of Metropolitan Hospital of Athens, Athens, Greece; A’ Robotic and Minimal Invasive General Surgery Department of Metropolitan Hospital of Athens, Athens, Greece; Department of Surgical Anatomy, School of Medicine, National and Kapodistrian University of Athens, Athens, Greece

## Abstract

Bochdalek hernias are usually diagnosed in newborns. However, they can occur in adults. Few reports exist regarding robotic repair of such hernias. We present a case of a female patient with symptomatic Bochdalek hernia, including the spleen. Patient was successfully treated by robotic-assisted surgical mesh with the use of indocyanine green (ICG). An 80-year-old female patient presented with dyspnea, angina and intermittent abdominal pain. She had loss of appetite and 15-kg weight loss within 3 months. Past medical history was unremarkable. Imaging revealed a left Bochdalek hernia. The patient underwent robotic-assisted surgery; hernia contents included stomach, parts of colon, omentum and remarkably the spleen. Sac was dissected free. Patency of organs was assessed with ICG. Diaphragmatic defect was repaired with mesh. Bochdalek hernias should be surgically repaired. Minimally invasive therapy is safe and effective. Intraoperative ICG use can provide excellent results with favorable clinical outcomes.

## INTRODUCTION

Bochdalek hernia is the most common type of congenital diaphragmatic hernias (CDHs) where the hernial sac protrudes through the foramen of Bochdalek; a hiatus created in the lumbocostal triangle by weakened diaphragmatic tissue on its posterior attachments. The etiology of such hernia is the failure of pleuroperitoneal membrane closure *in utero*, thus intra- and retroperitoneal components are able to prolapse within the hernial sac in the thorax [1]. Bochdalek and Morgagni hernias are considered to be two well-described congenital hernias that are primarily diagnosed in infant patients or young children. The former accounts for more than 95% of the CDHs, and the latter accounts for less than 5% of CDHs [2, 3]. A posterolateral CDH was first described in 1754 by McCauley and was furthermore studied and elaborated by the Czech pathologist Vincenz Alexander Bochdalek in 1848 [4, 5]. The incidence of Bochdalek hernia in newborns and young infants is ~1/2500 childbirths [6]. Dyspnea and cyanosis are the principal presenting symptoms. It can remain undiagnosed in cases where the hernia is rather small in size and consequently does not affect nor present with any symptomatology in the newborn [7, 8]. Various atypical and misleading symptoms make the diagnosis of a CDH in adults quite challenging [9]. Only few cases have been reported in medical literature regarding the occurrence of left Bochdalek hernia containing the spleen in adult population, which has been repaired via robotic surgical mesh repair [10, 11]. We present an interesting case of a 79-year-old female patient with left Bochdalek hernia where the hernial sac contained segments of large intestine, stomach, omentum and unexpectedly the spleen. She was successfully treated via minimal invasive robotic-assisted mesh repair, with intraoperative use of indocyanine green (ICG) and with the utilization of the firefly camera which is incorporated in the DaVinci Xi Robotic Surgical System.

## CASE REPORT

**Figure 1:**
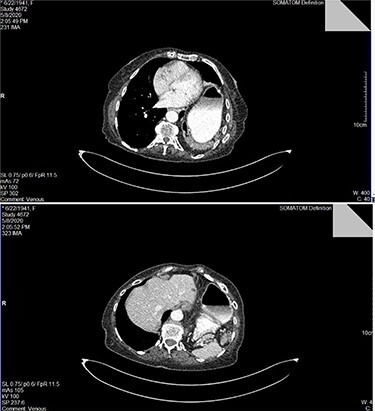
Top image: identification of the dilated stomach and segment of intestine in the left hemithorax in CT scan with intravenous contrast media; bottom image: identification of the spleen posteriorly to the dilated stomach within the right hemithorax.

A 79-year-old female patient, referred to the outpatient surgical department, presenting with acute onset of dyspnea during minor exercise, fatigue, angina and diffuse intermittent abdominal pain. She mentioned loss of appetite and more than 15-kg weight loss during a 3-month span. Her past medical history was unremarkable. She claimed no systematic use of medication. Her vital signs and electrocardiogram (ECG) had no pathological findings. Clinical examination revealed diffuse abdominal pain during deep palpation, reduced lung sounds and presence of bowel sounds in the left hemithorax and tympanic sound during percussion ipsilaterally. Blood sampling for a full blood count and biochemistry was performed. Results appeared to be within normal range, except from a slight platelet count (PLT) elevation (583 K/μl). Chest X-ray revealed the presence of large intestine and gastric bubble within the left hemithorax. Full cardiologic workup was conducted, with no pathological findings. A contrast-enhanced computed tomography (CT) of the chest and abdomen was performed. A left hemidiaphragm elevation was revealed, with part of the stomach, transverse and left colon and spleen herniating in the left chest trough a left posterior diaphragmatic defect. All signs were compatible with a Bochdalek hernia (Fig. 1).

Patient was subjected to a robotic-assisted mesh surgical repair. Four-trocar setup was used: one 8-mm trocar was placed above the umbilicus (optic camera), two 8-mm working ports were placed in the right- and left-midclavicular lines above the umbilicus, respectively, and one 5-mm port was placed at the left axillary line at the level of the umbilicus for the first assistant. The patient was placed in a steep reverse Trendelenberg position (35°).

During the first assessment of the abdominal cavity utilizing the optic camera, two remarkable findings were revealed. The heart and pericardium shifted to the contralateral side of the thorax due to compression from the hernial sac. This was depicted clearly via the pulsating heart over the right diaphragm. Additionally, an anatomical variation was encountered at the diaphragmatic border of the liver, where a steep ridge was formed at the area where the heart was beating and was chronically compressing the liver parenchyma, forming a pocket in the hepatic tissue.

**Figure 2:**
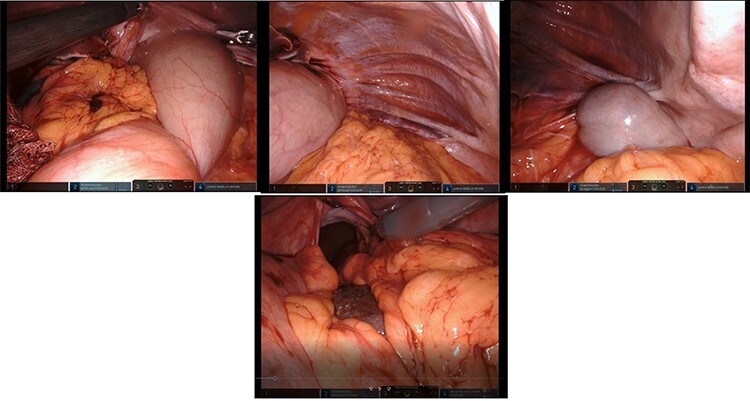
Top left: reduction of the dilated stomach from the hernial sac; retraction of the anterior border of the hiatus; top middle: presence and reduction of omentum from the hernial sac; top right: identification of the large intestine within the Bochdalek hernia; bottom middle: reduction of the spleen and great visualization of the large hiatus.

**Figure 3:**
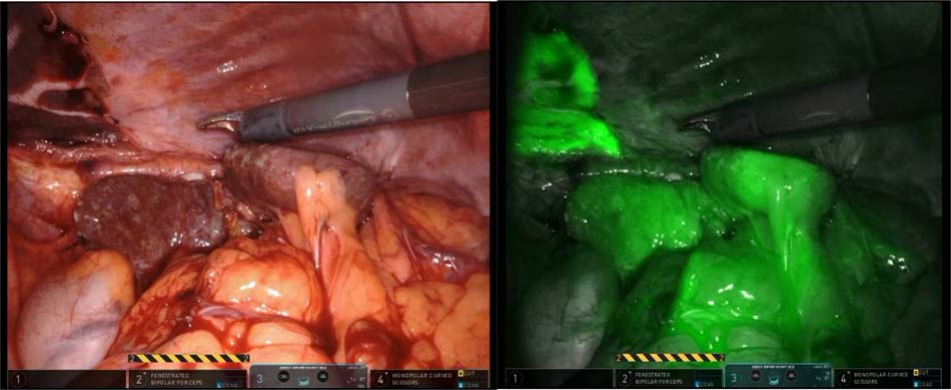
The use of intraoperative ICG and the use of Firefly camera to assess blood supply and viability of the spleen.

**Figure 4:**
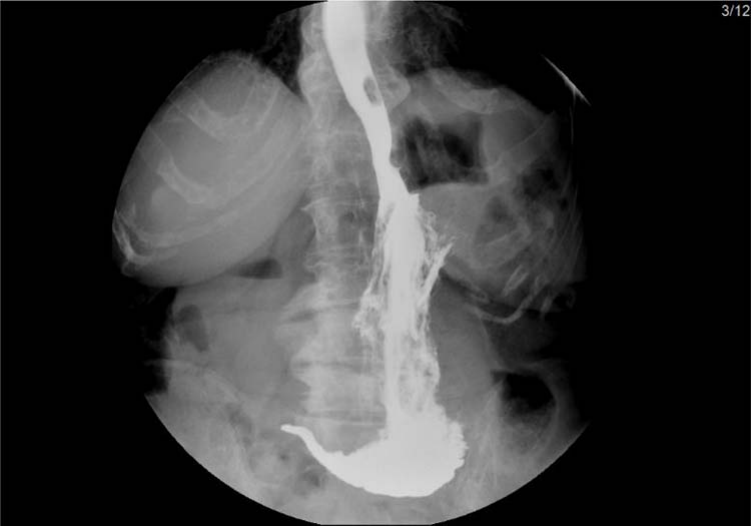
Upper GI fluoroscopy to assess the results of the hernia repair during the third POD.

After mobilization of the left liver lobe, visualization of the hiatus was achieved. A two silk suture was placed through the avascular hepatogastric ligament and outside the patient’s anterior abdomen with an extracorporeal knot in order to assure atraumatic elevation-retraction of the liver. Space was created in the surgical field due to the absence of a retracting surgical instrument within the abdominal cavity. The hernia’s sac was then dissected with use of energy device and bipolar cautery with minimal traumatic tissue handling; the herniated stomach, transverse and left colon, omentum and finally spleen were dissected free (Fig. 2). Due to the atypical clinical symptomatology as well as increased PLT, the suspicion of chronic ischemia of the herniated organs was raised. ICG was administered to assess the patency of blood supply in the herniated organs and specially to the spleen; blood supply was excellent and as a result, no further resections were needed (Fig. 3). An 8- × 4-cm posterolateral diaphragmatic defect was revealed; the defect was repaired with the use of ProGrip mesh and was supported by a continuous V-loc suture for fixation. The patient resuscitated from anesthesia in great condition and returned to the ward, with an uneventful course. A post-operative upper GI fluoroscopy was conducted at the second post-operative day (POD), and oral diet was commenced (Fig. 4).

An episode of paroxysmal supraventricular tachycardia appeared during the third POD. The arrhythmia was controlled spontaneously. The patient was discharged from the hospital during the fifth POD. Patient is followed-up for 1 year with no signs of recurrence.

## DISCUSSION

Leftside Bochdalek hernia is a well-described and common entity in literature (accounts for 85% of CDH). A general older consensus is that, in order to achieve greater visualization and freedom of movement in such cases, a full laparotomy, laparoscopy and thoracoscopy or merely thoracoscopic approach was more favorable [12]. Traditional open approach is nowadays rarely selected and has been substituted by either laparoscopic or thoracoscopic repair. The reasoning is that minimally invasive techniques have lowered the average hospital stay dramatically (4 vs. 14 days), have almost effaced 30-day morbidity rate and have narrowed down complication rates (5 vs. 17%) compared to open repairs [13].

In leftside Bochdalek hernias, retraction of the left liver lobe can be challenging and additional surgical instruments are deployed to achieve that. In this case report, a unique technique in order to retract the liver is used (two silk suture with extracorporeal knot). The goal is to liberate the surgeons’ hands and create a spacious surgical field.

It is common that LBCDH can stay undiagnosed in young age and present with varying symptomatology in late adulthood [7, 8]. In our case, the most prominent presenting symptoms were nausea, vomiting, loss of appetite, left quadrant and epigastric pain and dyspnea. Absence of any traumatic episode in the past medical history was clearly stated. Regardless of the symptomatology, surgical treatment is suggested in such situations in order to avoid common life-threatening complications of herniation (i.e. strangulation and ischemia) as well as for avoiding unnecessary cardiopulmonary burden through compressing phenomena. The goal of the operation is to identify the hiatus of the hernia, reduce the sac and its contents and repair the hernia by means of synthetic mesh or via primary closure. The effectiveness of each in robotic surgery is still unclear. In a recent study, it is reported that the use of mesh is more common than primary repair in original laparoscopy [13]. The use of mesh appears to have excellent results in terms of avoiding recurrence while achieving excellent support of the tissue. On the other hand, primary closure in small defects is preferred by some surgeons when using the Davinci Robotic System due to the ability of free rotation and movement of the surgeon’s hand and substantially more precise suturing ability.

The use of ICG while utilizing the Firefly camera of the DaVinci Xi Robotic System is another innovation used in this case. It is the only case reported of leftside Bochdalek hernia in international literature, which utilizes such contrast media intraoperatively. Assessment of blood supply with a harmless substance at the time of operation is an undeniable weapon in the surgeon’s arsenal in order to provide the patient with the optimal treatment without inadvertently assuming false blood supply of the herniated organs. The phenomenon of ischemia in such hernias has been reported and further anatomical dissections were required [10, 14, 15]. Macroscopic alteration of blood supply could be absent at time of operation, so intraoperative ICG green application readily evaluates such cases.

## CONCLUSION

Leftsided Bochdalek hernias are the most common CDHs, most usually occurring in newborns and young children. However, in a substantial amount of cases, they remain undiagnosed. Symptoms can appear for the first time in late adulthood as the hiatus increases in size. Notwithstanding of clinical presentation, Bochdalek hernias should be surgically repaired. Minimally invasive therapy is feasible, safe and effective. Robotic-assisted repair provides all the advances of robotic surgery, which lead to reduced hospitalization time.
